# The Interaction of Dietary Pectin, Inulin, and Psyllium with Copper Nanoparticle Induced Changes to the Cardiovascular System

**DOI:** 10.3390/nu15163557

**Published:** 2023-08-11

**Authors:** Michał Majewski, Leszek Gromadziński, Ewelina Cholewińska, Katarzyna Ognik, Bartosz Fotschki, Jerzy Juśkiewicz

**Affiliations:** 1 Department of Pharmacology and Toxicology, Faculty of Medicine, University of Warmia and Mazury in Olsztyn, 10-082 Olsztyn, Poland; 2 Department of Cardiology and Internal Medicine, Faculty of Medicine, University of Warmia and Mazury in Olsztyn, 10-082 Olsztyn, Poland; leszek.gromadzinski@uwm.edu.pl; 3 Department of Biochemistry and Toxicology, Faculty of Animal Sciences and Bioeconomy, University of Life Sciences in Lublin, 20-950 Lublin, Poland; ewelina.cholewinska@up.lublin.pl (E.C.); kasiaognik@poczta.fm (K.O.); 4 Division of Food Science, Institute of Animal Reproduction and Food Research, Polish Academy of Sciences, 10-748 Olsztyn, Poland; b.fotschki@pan.olsztyn.pl

**Keywords:** 1400W, cyclooxygenase, GAPDH, heme oxygenase-1, iCAM-1, nitric oxide synthase, NS-398, SC-560

## Abstract

We aimed to analyze how supplementation with a standard (recommended, 6.5 mg/kg) or enhanced (two-times higher, 13 mg/kg) dose of copper (Cu), in the form of nanoparticles (NPs) along with dietary intervention via the implementation of diverse types of fiber, affects the cardiovascular system in rats. Nine-week-old male Wistar Han rats (n/group = 10) received, for an additional 6 weeks, a controlled diet with cellulose as dietary fiber and ionic Cu (in the form of carbonate salt). The experimental groups received cellulose, pectin, inulin, and psyllium as dietary fiber, together with CuNPs (6.5 or 13 mg/kg diet). After the experimental feeding, samples of blood, hearts, and thoracic arteries were collected for further analysis. Compared to pectin, and under a standard dose of CuNPs, inulin and psyllium beneficially increased the antioxidant capacity of lipid- and water-soluble compounds in the blood, and decreased heart malondialdehyde. Moreover, pectin decreased heart catalase (CAT) and cyclooxygenase (COX)-2 in the aortic rings compared to inulin and psyllium under standard and enhanced doses of copper. When the dose of CuNPs was enhanced, inulin and psyllium potentiated vasodilation to acetylcholine by up-regulation of COX-2-derived vasodilator prostanoids compared to both cellulose and pectin, and this was modulated with selective inducible nitric oxide synthase (iNOS) inhibitor for psyllium only. Moreover, inulin decreased heart CAT compared to psyllium. Our results suggest that supplementation with dietary fiber may protect the vascular system against potentially harmful metal NPs by modulating the antioxidant mechanisms.

## 1. Introduction

Copper (Cu) is an essential trace element for proper body-building processes. Thus, to maintain the functioning of the organism, it is important to provide an adequate Cu intake with a daily diet, bearing in mind uncertainties regarding Cu concentration in various food products and drinking water [1]. However, very often, a daily diet is enriched with ionic forms of Cu to fulfill daily recommendations and to improve Cu status in order to reduce the risk of cardiovascular disease and atherosclerosis, which are attributed to Cu deficiency [2]. Cytopenia and neuropathy are caused by Cu deficiency, which can be treated by Cu supplementation. Alcohol abuse, zinc ingestion, long-term total parenteral nutrition, and intestinal resection are well-known risk factors for Cu deficiency [3].

Metal and metal oxide nanoparticles were recently introduced into the daily diet of animals due to the specific properties of nano-sized materials. This was undertaken in order to reduce the daily dose of minerals and to increase their bioavailability. However, metal NPs carry a possible risk that outstate the benefits of ionic forms due to the potential toxicity of NPs themselves. Only a few recent studies are partly consistent with safety reports, in contrast to many others which point towards the intensification of lipid peroxidation and impairment of antioxidant defense mechanisms [4].

A daily diet rich in fiber, including fruits, vegetables, cereals, and nuts, has a positive effect on health, since their consumption has been related to decreased cardiovascular disease, gut motility, colonic health, and risk for colorectal carcinoma [5,6]. In general, dietary fiber is divided into water-soluble and insoluble forms. Cellulose, hemicellulose, and lignin are not soluble in water, whereas sticky pectin, swelling psyllium, and non-viscous inulin (with prebiotic importance) change their properties in water. On the other hand, the consumption of dietary fiber may affect the absorption of nutrients, including minerals and heavy metals, in a different way [7,8,9]. Increased zinc and molybdenum intake may lead to Cu deficiency disorders [10]. Other dietary factors, such as fiber, carbohydrates, and vitamin C, also decrease the bioavailability of Cu [11]. Dietary fiber increases the acidity of the intestine, which affects Cu absorption [11]. Dietary pectin and intact cellulose have no effect on the Cu concentration, which is contrary to hemicellulose, which decreases Cu balance [12].

Available data indicate that the bioavailability of Cu is decreased by different dietary forms of fiber; hence, we assumed that the combination of CuNPs with a different type of dietary fiber would affect the absorption and modulate the physiological processes of the body. We aimed to verify the hypothesis that dietary fiber in the form of cellulose, pectin, inulin, and psyllium will have beneficial effects on the antioxidant system, which will beneficially modify blood plasma, heart, and vasculature function in rats subjected to CuNPs under the standard (6.5 mg/kg) and two-times higher (13.0 g/kg) doses of Cu.

## 2. Materials and Methods

### 2.1. Drugs and Chemicals

Acetylcholine (chloride), noradrenaline (hydrochloride), 1400W (N-(3-(Aminomethyl)benzyl)acetamidine), NS-398 (N-[2-(Cyclohexyloxy)-4-nitrophenyl]methanesulfonamide), and SC-560 (5-(4-Chlorophenyl)-1-(4-methoxyphenyl)-3-trifluoromethyl pyrazole) were obtained from Sigma-Aldrich (St. Louise, MO, USA). Copper as carbonate (purity ≥ 99%) was obtained from Poch (Gliwice, Poland). Stock solutions (10 mM) of these drugs were prepared in distilled water, except for noradrenaline, which was dissolved in NaCl + ascorbic acid (0.9% + 0.01% *w*/*v*) solution; NS-398 and SC-560 were dissolved in DMSO; and 1400W in methanol. The solvent concentration was less than 0.01% (*v*/*v*). These solutions were stored at −20 °C, and appropriate dilutions were made in Krebs–Henseleit solution (KH in mM: NaCl 115; CaCl_2_ 2.5; KCl 4.6; KH_2_PO_4_ 1.2; MgSO_4_ 1.2; NaHCO_3_ 25; glucose 11.1) on the day of the experiment.

CuNPs (as 99.9% purity powder, of 40–60 nm size, 12 m^2^/g SSA, spherical morphology, 0.19 g/cm^3^ bulk density, 8.9 g/cm^3^ true density) were purchased from Sky Spring Nanomaterials (Sky Spring Nanomaterials Inc., Houston, TX, USA). The zeta potential and the size were previously measured [13].

### 2.2. Experimental Protocol and Procedures

The 6-week experiment was conducted on outbred 9-week-old male Wistar rats (Cmdb:Wi CMDB). An in vivo protocol was compiled according to the European Convention for the Protection of Vertebrate Animals used for Experimental and other Scientific Purposes, Directive 2010/63/EU for animal experiments, and was approved by the Local Ethics Committee for Animal Experiments. The details of an experimental schema were provided previously [14]. In brief, the control (C) animals (n/group = 10) received, for an additional 6 weeks, a controlled diet, with 8% cellulose as dietary fiber and ionic Cu in the form of carbonate salt. The experimental groups received either 8% cellulose (CN), 6% pectin (PN), 6% inulin (JN), or 6% psyllium (SN) as dietary fiber, together with CuNPs (6.5 or 13 mg/kg). The dietary pectin, inulin, and psyllium were coupled with 2% cellulose in order to keep the dietary fiber dose. 

This study was conducted on two main groups:

(A) Control, which was further subdivided into:
  1. Control C (8% cellulose and 6.5 mg/kg of ionic Cu)
  2. Control CH (8% cellulose and 13 mg/kg of ionic Cu)
(B) Supplemented, which was further subdivided into:
  1. CN (8% cellulose and 6.5 mg/kg of CuNPs)
  2. CNH (8% cellulose and 13 mg/kg of CuNPs)
  3. PN (6% pectin and 6.5 mg/kg of CuNPs)
  4. PNH (6% pectin and 13 mg/kg of CuNPs)
  5. JN (6% inulin and 6.5 mg/kg of CuNPs)
  6. JNH (6% inulin and 13 mg/kg of CuNPs)
  7. SN (6% psyllium and 6.5 mg/kg of CuNPs)
  8. SNH (6% psyllium and 13 mg/kg of CuNPs)

The rats were housed individually in stainless-steel cages under a stable temperature (22 ± 1 °C), relative humidity 60 ± 5%, a 12 h light-dark cycle, and a ventilation rate of 15 air changes per hour. For 6 weeks, the rats had free access to tap water and diets, which were prepared and then stored at 4 °C in hermetic containers until the end of the experiment. The rats were fasted for 12 h and then were anesthetized by intraperitoneal injection of ketamine + xylazine (100 mg/kg +10 mg/kg of body weight). The blood was collected from the caudal vena cava of anesthetized animals. 

### 2.3. Blood Plasma Analysis

The antioxidant capacity of water (ACW)- and lipid (ACL)-soluble compounds (in µg/mL) was determined with Photochem (Analytik Jena AG, Jena, Germany). This photo-chemiluminescence method of detection generates free radicals that are removed with the antioxidants presented in blood plasma, and the remaining radicals are quantified. The calibration curve was prepared with ascorbic acid and Trolox as standards for ACW and ACL [15].

### 2.4. Markers of Antioxidant Status in Aortic Rings

To determine concentrations of oxidative stress markers in the aortic rings, commercial ELISA Kits were used according to the manufacturer’s instructions (Table S1). Cyclooxygenase 1 (COX-1), cyclooxygenase 2 (COX-2), heme oxygenase-1 (HO-1), endothelial nitric oxide synthase 3 (NOS3), glyceraldehyde 3-phosphate dehydrogenase (GAPDH), and intercellular adhesion molecule 1 (ICAM-1) were examined. The absorbance in the ELISA test plate was measured by Thermo Scientific microplate readers (Varioskan LUX, Bremen, Germany) at a wavelength of λ = 450 nm.

### 2.5. Markers of Antioxidant Status in the Heart

Heart malondialdehyde (MDA, µmol/kg) reacts with thiobarbituric acid (TBA), which gives an MDA–TBA adduct, which was quantified with a fluorometric assay kit (ab118970) at Ex/Em = 532/553 nm. CAT activity was determined with an Oxis International, Inc., (Portland, OR, USA) diagnostic kit following the manufacturer’s instructions. SOD activity was determined with Ransel and Ransod colorimetric diagnostic kits from Randox (Warsaw, Poland) following the manufacturer’s instructions.

### 2.6. Vascular Reactivity Studies

Aortic rings from the thoracic aorta (4–5 mm length) were aerated with a carbogen gas in stagnant 5 mL chambers (Graz, Barcelona, Spain) throughout the entire experiment. Two parallel stainless-steel wires, one fixed to the bath wall and the other connected to a force transducer, were implemented through the lumen of the aortic rings with a pre-load tension of 1 g (FT20, TAM-A, Hugo Sachs Elektronik, March, Germany). We used high KCl (75 mM) and acetylcholine (10 µM) to check the functional integrity of aortic rings. Further, aortic rings were pre-incubated for 30 min with either the selective cyclooxygenase-2 (COX-2) inhibitor (NS-398) at 10 µM, the selective cyclooxygenase-1 (COX-1) inhibitor (SC-560) at 10 µM plus NS-398, or the inducible nitric oxide synthase (iNOS) inhibitor (1400 W) at 1 µM, and contracted with noradrenaline (0.1 µM). After the incubation period, the cumulative concentrations of acetylcholine (0.1 nM–10 µM) were added into the incubation chambers.

### 2.7. Retrograde Heart Perfusion: The Langendorff Heart

After excision, hearts were weighed and inserted in a Langendorff system and monitored with the ISOHEART software 73-0161 (Hugo Sachs Elektronik, March, Germany). Diastolic pressure was set to 8–10 mmHg by inflating the balloon. Systolic (mmHg), diastolic (mmHg), mean arterial pressure (mmHg), and heart rate (beats/min) were recorded. 

### 2.8. Data Analysis and Statistics

Results are presented as means ± SEM (for vascular reactivity studies) and means ± SD (for other data) and were prepared in GraphPad Prism 10.0. Vascular relaxation to acetylcholine was calculated as a percentage of the contraction to NA. The group comparison was performed by two-way ANOVA with an appropriate post hoc test. Differences were considered significant when *p* ≤ 0.05.

## 3. Results

### 3.1. The Antioxidant Capacity of Blood Plasma

#### 3.1.1. Blood Plasma ACW

**Figure 1:**
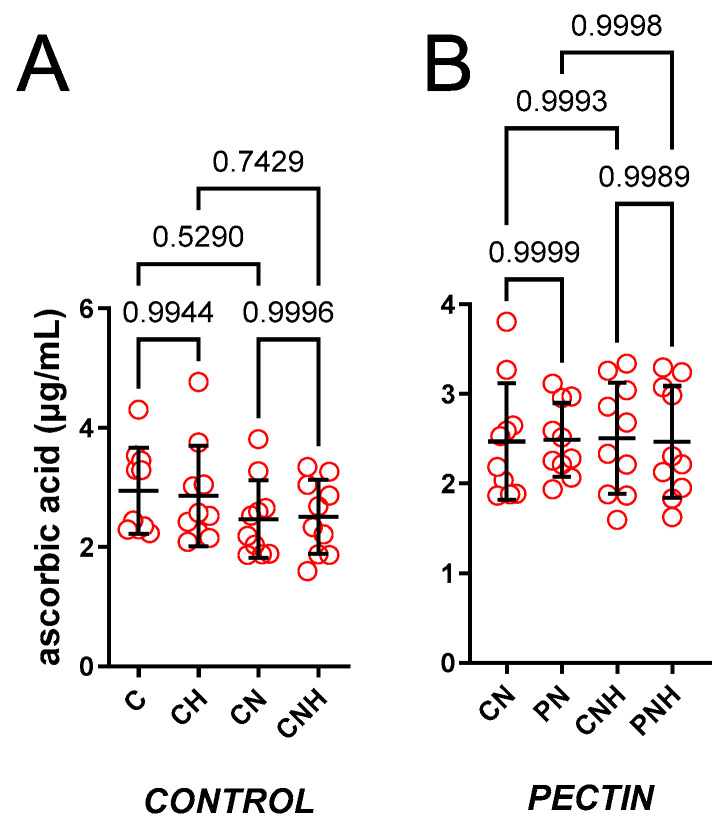

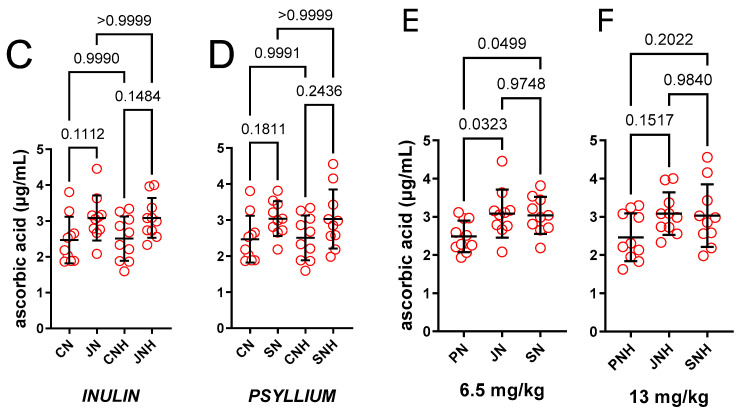
Blood plasma ACW measured in rats fed with experimental diets. Dietary treatments: rats from groups C and CH were fed a control diet with a standard or enhanced copper content (6.5 or 13 mg/kg as CuCO_3_) together with cellulose (8%) as a source of dietary fiber (**A**); group CN and CNH were supplemented with CuNPs (6.5 or 13 mg/kg) together with cellulose (8%) as a source of dietary fiber (**A**); group PN and PNH were supplemented with CuNPs (6.5 or 13 mg/kg) together with cellulose (2%) and pectin (6%) as a source of dietary fiber (**B**); group JN and JNH were supplemented with CuNPs (6.5 or 13 mg/kg) together with cellulose (2%) and inulin (6%) as a source of dietary fiber (**C**); group SN and SNH were supplemented with CuNPs (6.5 or 13 mg/kg) together with cellulose (2%) and psyllium (6%) as a source of dietary fiber (**D**); a comparison of dietary fiber in regard to a standard (6.5 mg/kg) (**E**); and enhanced (13 mg/kg) copper dose from CuNPs (**F**). Values are means ± SD, n = 10, *p* ≤ 0.05 (two-way ANOVA with Tukey’s multiple comparisons test). ACW, antioxidant capacity of water-soluble compounds. Compared to pectin (PN), and under a standard dose of CuNPs, inulin (JN) and psyllium (SN) increased ACW in the blood plasma. Red circle is the single value measured from one animal.

The effect of a standard or an enhanced dose of nano-Cu (6.5 or 13 mg/kg) was insignificant, see Figure 1A.

The effect of decreased cellulose content and high CuNP dose was insignificant, see Figure 1B–D.

The effect of PN vs. JN vs. SN and a high CuNP dose. JN (1.24-fold) and SN (1.22-fold) increased ACW compared to PN under a standard dose of CuNPs, see Figure 1E. Enhanced doses of CuNPs made this insignificant (1.25-fold and 1.23-fold), see Figure 1F.

#### 3.1.2. Blood Plasma ACL

**Figure 2:**
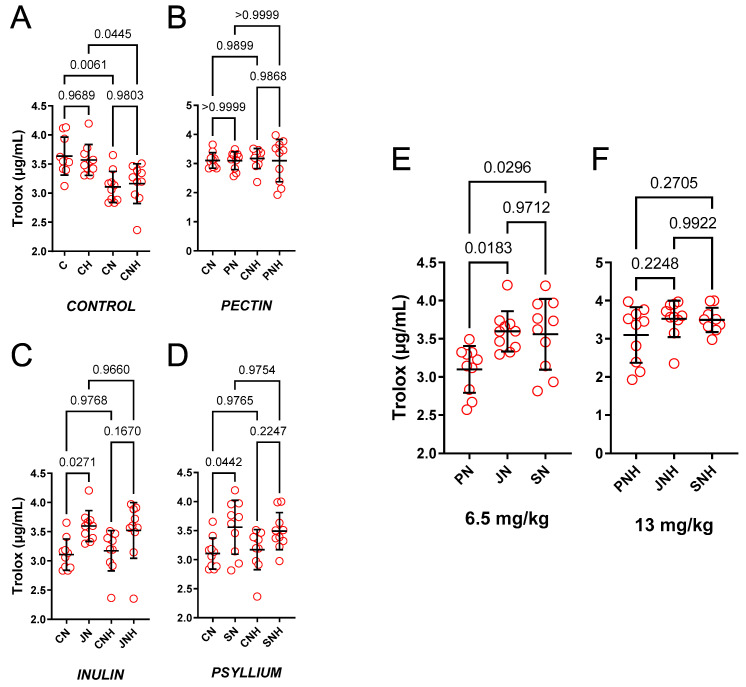
Blood plasma ACL measured in rats fed with experimental diets. Dietary treatments: rats from groups C and CH were fed a control diet with a standard or enhanced copper content (6.5 or 13 mg/kg as CuCO_3_) together with cellulose (8%) as a source of dietary fiber (**A**); group CN and CNH were supplemented with CuNPs (6.5 or 13 mg/kg) together with cellulose (8%) as a source of dietary fiber (**A**); group PN and PNH were supplemented with CuNPs (6.5 or 13 mg/kg) together with cellulose (2%) and pectin (6%) as a source of dietary fiber (**B**); group JN and JNH were supplemented with CuNPs (6.5 or 13 mg/kg) together with cellulose (2%) and inulin (6%) as a source of dietary fiber (**C**); group SN and SNH were supplemented with CuNPs (6.5 or 13 mg/kg) together with cellulose (2%) and psyllium (6%) as a source of dietary fiber (**D**); a comparison of dietary fiber in regard to a standard (6.5 mg/kg) (**E**); and enhanced (13 mg/kg) copper dose from CuNPs (**F**). Values are means ± SD, n = 10, *p* ≤ 0.05 (two-way ANOVA with Tukey’s multiple comparisons test). ACL, antioxidant capacity of lipid-soluble compounds. CuNPs under recommended 6.5 mg/kg and two-times higher dose decreased ACL. Compared to cellulose (CN) and pectin (PN), and under standard doses of CuNPs, inulin (JN) and psyllium (SN) increased ACL in the blood plasma.

Both CN (0.85-fold) and CNH (0.89-fold) decreased ACL versus respective controls (C and CH), see Figure 2A.

*The effect of decreased cellulose content and high CuNP dose.*

Only JN (1.16-fold) and SN (1.15-fold) increased ACL compared to CN. Enhanced doses of CuNPs made this insignificant (1.11-fold and 1.10-fold, respectively), see Figure 2B–D.

*The effect of PN* vs. *JN vs. SN and a high CuNP dose.*

JN (1.16-fold) and SN (1.15-fold) increased ACL compared to PN, see Figure 2E. Enhanced doses of CuNPs made this insignificant (1.14-fold and 1.13-fold, respectively), see Figure 2F.

### 3.2. Heart Examination

#### 3.2.1. Heart Malondialdehyde

**Figure 3:**
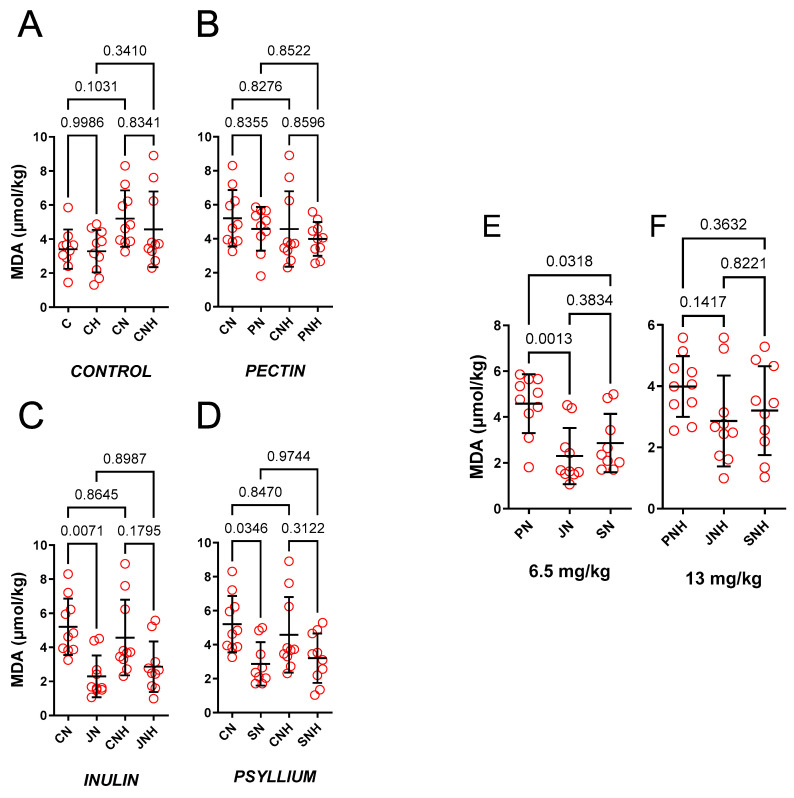
Malondialdehyde measured in the hearts of rats fed with experimental diets. Dietary treatments: rats from groups C and CH were fed a control diet with a standard or enhanced copper content (6.5 or 13 mg/kg as CuCO_3_) together with cellulose (8%) as a source of dietary fiber (**A**); group CN and CNH were supplemented with CuNPs (6.5 or 13 mg/kg) together with cellulose (8%) as a source of dietary fiber (**A**); group PN and PNH were supplemented with CuNPs (6.5 or 13 mg/kg) together with cellulose (2%) and pectin (6%) as a source of dietary fiber (**B**); group JN and JNH were supplemented with CuNPs (6.5 or 13 mg/kg) together with cellulose (2%) and inulin (6%) as a source of dietary fiber (**C**); group SN and SNH were supplemented with CuNPs (6.5 or 13 mg/kg) together with cellulose (2%) and psyllium (6%) as a source of dietary fiber (**D**); a comparison of dietary fiber in regard to a standard (6.5 mg/kg) (**E**); and enhanced (13 mg/kg) copper dose from CuNPs (**F**). Values are means ± SD, n = 10, *p* ≤ 0.05 (two-way ANOVA with Tukey’s multiple comparisons test). MDA, malondialdehyde. Compared to cellulose (CN) and pectin (PN), and under standard doses of CuNPs, inulin (JN) and psyllium (SN) decreased malondialdehyde in hearts.

The effect of a standard or an enhanced dose of nano-Cu (6.5 and 13 mg/kg) was insignificant, see Figure 3A. However, CN exhibit tendency to increase MDA.

*The effect of decreased cellulose content and high CuNP dose.*

Only JN (0.44-fold) and SN (0.55-fold) decreased MDA compared to CN. Enhanced doses of CuNPs made this insignificant (0.63-fold and 0.70-fold, respectively), see Figure 3B–D.

*The effect of PN vs. JN vs. SN and a high CuNP dose.*

JN (0.50-fold) and SN (0.62-fold) decreased MDA compared to PN, see Figure 3E. Enhanced doses of CuNPs made this insignificant (0.72-fold and 0.80-fold, respectively), see Figure 3F.

#### 3.2.2. Heart SOD

Heart SOD was neither changed by the dietary fiber nor by the enhanced CuNP dose (data not presented).

#### 3.2.3. Heart CAT

*The effect of nano-Cu (6.5 and 13 mg/kg).*

**Figure 4:**
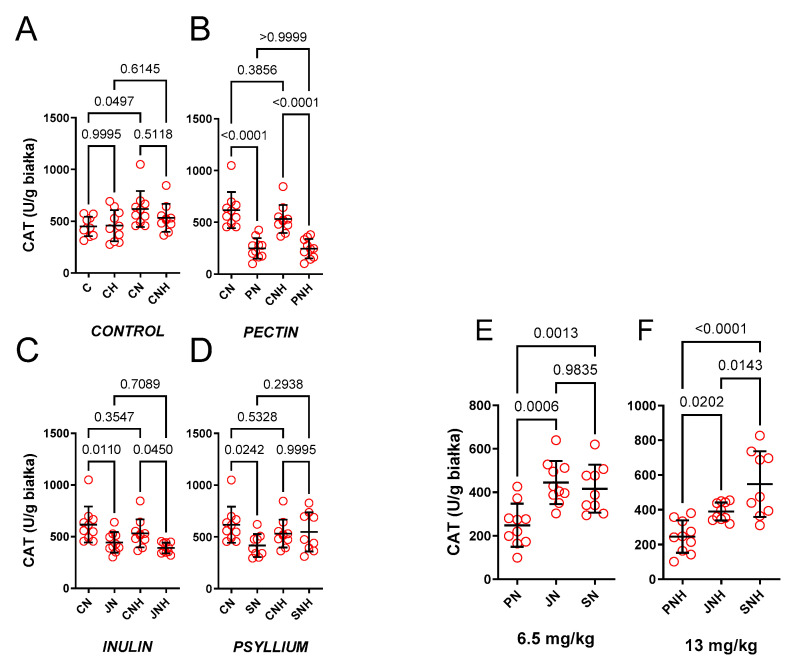
Catalase measured in the hearts of rats fed with experimental diets. Dietary treatments: rats from groups C and CH were fed a control diet with a standard or enhanced copper content (6.5 or 13 mg/kg as CuCO_3_) together with cellulose (8%) as a source of dietary fiber (**A**); group CN and CNH were supplemented with CuNPs (6.5 or 13 mg/kg) together with cellulose (8%) as a source of dietary fiber (**A**); group PN and PNH were supplemented with CuNPs (6.5 or 13 mg/kg) together with cellulose (2%) and pectin (6%) as a source of dietary fiber (**B**); group JN and JNH were supplemented with CuNPs (6.5 or 13 mg/kg) together with cellulose (2%) and inulin (6%) as a source of dietary fiber (**C**); group SN and SNH were supplemented with CuNPs (6.5 or 13 mg/kg) together with cellulose (2%) and psyllium (6%) as a source of dietary fiber (**D**); a comparison of dietary fiber in regard to a standard (6.5 mg/kg) (**E**); and enhanced (13 mg/kg) copper dose from CuNPs (**F**). Values are means ± SD, n = 10, *p* ≤ 0.05 (two-way ANOVA with Tukey’s multiple comparisons test). CAT, catalase. Under a standard dose of CuNPs, experimental feeding (CN) increased CAT. Compared to cellulose (CN), and under a standard dose of CuNPs, pectin (PN), inulin (JN) and psyllium (SN) decreased CAT. When the dose of CuNPs was enhanced, pectin (PN) and inulin (JN), but not psyllium (SN), decreased CAT. Under standard and enhanced doses of CuNPs, pectin (PN and PNH CuNPs) decreased CAT compared to inulin (JN and JNH) and psyllium (SN and SNH). Under an enhanced CuNPs dose, a significant difference was observed between inulin (JNH) and psyllium (SNH).

Only CN (1.37-fold) increased CAT versus the respective control (C). The effect of CNH was insignificant (1.16-fold), see Figure 4A.

*The effect of decreased cellulose content and high CuNP dose.*

PN (0.40-fold), JN (0.72-fold), and SN (0.67-fold) decreased CAT compared to CN. Enhanced doses of CuNPs made this insignificant only for SNH (0.97-fold). For PNH and JNH, it remained significant (0.46-fold and 0.73-fold, respectively), see Figure 4B–D.

*The effect of PN* vs. *JN vs. SN and a high CuNP dose.*

Standard and enhanced doses of CuNPs decreased much more CAT for PN vs. JN (0.56-fold) and PN vs. SN (0.60-fold), as well as PNH vs. JNH (0.63-fold) and PNH vs. SNH (0.45-fold), see Figure 4E. Moreover, CAT was further decreased for JNH compared to SNH (0.71-fold), and this was not observed for JN compared to SN (1.1-fold), see Figure 4F.

#### 3.2.4. Retrograde Heart Perfusion: The Langendorff Heart

Systolic, diastolic, mean arterial pressure, and heart rate were not modified (data not presented).

### 3.3. Vascular Studies

#### 3.3.1. Vascular Reactivity

The vasoconstrictor response to high KCl (75 mM) and noradrenaline (0.1 nM–10 µM) did not differ between the studied groups (data not presented).

The vasodilator response to sodium nitroprusside (exogenous NO donor), which is the endothelium independent mechanism, did not differ between the studied groups (data not presented).

**Figure 5:**
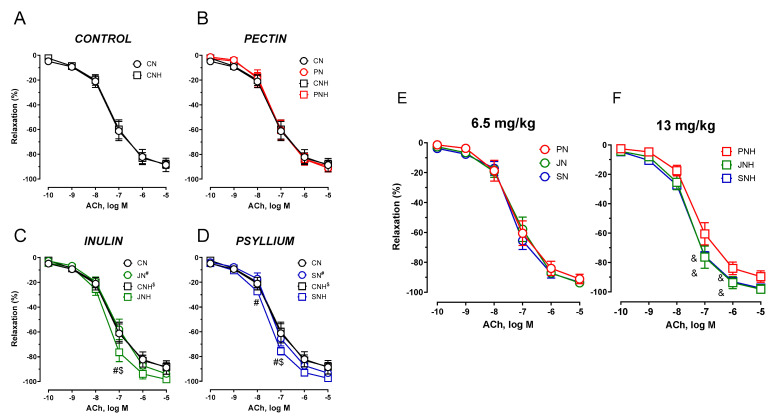
The cumulative concentration-response curves to acetylcholine in the isolated thoracic arteries dissected from rats fed with experimental diets. Dietary treatments: rats from groups C and CH were fed a control diet with a standard or enhanced copper content (6.5 or 13 mg/kg as CuCO_3_) together with cellulose (8%) as a source of dietary fiber (**A**); group CN and CNH were supplemented with CuNPs (6.5 or 13 mg/kg) together with cellulose (8%) as a source of dietary fiber (**A**); group PN and PNH were supplemented with CuNPs (6.5 or 13 mg/kg) together with cellulose (2%) and pectin (6%) as a source of dietary fiber (**B**); group JN and JNH were supplemented with CuNPs (6.5 or 13 mg/kg) together with cellulose (2%) and inulin (6%) as a source of dietary fiber (**C**); group SN and SNH were supplemented with CuNPs (6.5 or 13 mg/kg) together with cellulose (2%) and psyllium (6%) as a source of dietary fiber (**D**); a comparison of dietary fiber in regard to a standard (6.5 mg/kg) (**E**); and enhanced (13 mg/kg) copper dose from CuNPs (**F**). Values are means ± SEM, n = 10, ^#^ vs. standard CuNPs, ^$^ vs. cellulose, ^&^ vs. pectin, *p* ≤ 0.05 (two-way ANOVA with Tukey’s multiple comparisons test). Compared to cellulose (CNH) and pectin (PNH), and under enhanced doses of CuNPs, inulin (JNH) and psyllium (SNH) potentiated vasodilation to acetylcholine.

The vasodilator response to acetylcholine (ACh) is the endothelium dependent mechanism. The effect of a standard or an enhanced dose of CuNPs (6.5 and 13 mg/kg) was insignificant (data not presented). CNH did not modify the vasodilatory response to acetylcholine compared to CN, see Figure 5A.

*The effect of decreased cellulose content and high CuNP dose.*

An enhanced dose of CuNPs potentiated vasodilation for JNH and SNH vs. CNH. This was not observed for PNH. A significant difference was also observed between JNH vs. JN and SNH vs. SN, see Figure 5B–D.

*The effect of PN* vs. *JN vs. SN and a high CuNP dose.*

An enhanced dose of CuNPs potentiated vasodilation to acetylcholine under a JNH and SNH supplementation. That was not observed under the standard CuNP dose, see Figure 5E,F.

**Figure 6:**
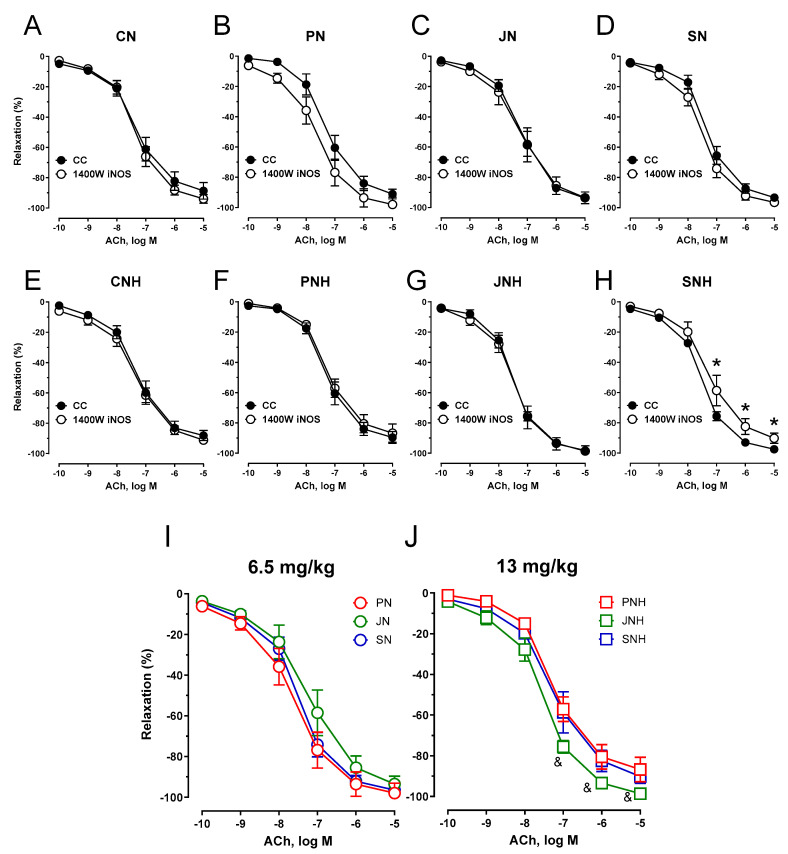
Effects of iNOS inhibition on the cumulative concentration-response curves to acetylcholine in the isolated thoracic arteries dissected from rats fed with experimental diets. Dietary treatments: rats from group CN (**A**) and CNH (**E**) were supplemented with CuNPs (6.5 or 13 mg/kg from CuNPs) together with cellulose (8%) as a source of dietary fiber; group PN (**B**) and PNH (**F**) were supplemented with CuNPs (6.5 or 13 mg/kg from Cu-nanoparticles) together with cellulose (2%) and pectin (6%) as a source of dietary fiber; group JN (**C**) and JNH (**G**) were supplemented with CuNPs (6.5 or 13 mg/kg from CuNPs) together with cellulose (2%) and inulin (6%) as a source of dietary fiber; group SN (**D**) and SNH (**H**) were supplemented with CuNPs (6.5 or 13 mg/kg from CuNPs) together with cellulose (2%) and psyllium (6%) as a source of dietary fiber. A comparison of dietary fiber in regard to a standard (6.5 mg/kg) (**I**); and enhanced (13 mg/kg) (**J**) copper dose from CuNPs. Values are means ± SEM, n = 10, * vs. control conditions, ^&^ vs. pectin, *p* ≤ 0.05 (two-way ANOVA with Tukey’s multiple comparisons test). Under an enhanced CuNP dose, the iNOS inhibition modified the response to acetylcholine for psyllium (SNH) compared to pectin (PNH) and the control conditions. This weakened the maximal response and shifted the curve to the right compared to control conditions.

Pre-incubation of the aortic rings with the inducible nitric oxide synthase (iNOS) inhibitor 1400 W shifted the curve to the right for SNH only. The vasodilator response to acetylcholine between the other studied groups was not modified (see Figure 6).

**Figure 7:**
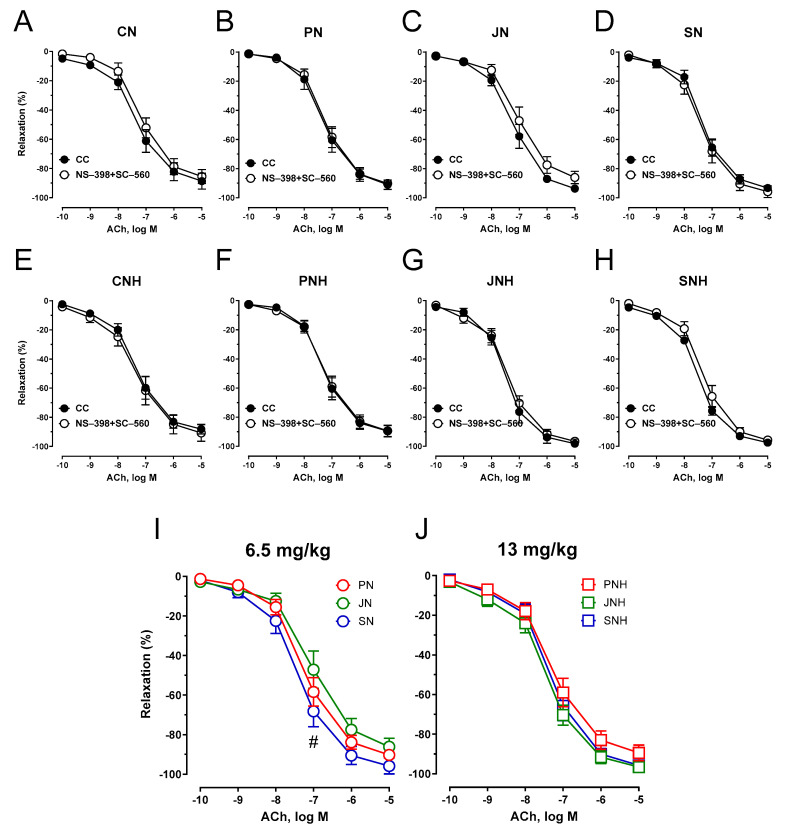
Effects of COX-1 and COX-2 inhibition on the cumulative concentration-response curves to acetylcholine in the isolated thoracic arteries dissected from rats fed with experimental diets. Dietary treatments: rats from group CN (**A**) and CNH (**E**) were supplemented with CuNPs (6.5 or 13 mg/kg from Cu-nanoparticles) together with cellulose (8%) as a source of dietary fiber; group PN (**B**) and PNH (**F**) were supplemented with CuNPs (6.5 or 13 mg/kg from Cu-nanoparticles) together with cellulose (2%) and pectin (6%) as a source of dietary fiber; group JN (**C**) and JNH (**G**) were supplemented with CuNPs (6.5 or 13 mg/kg from Cu-nanoparticles) together with cellulose (2%) and inulin (6%) as a source of dietary fiber; group SN (**D**) and SNH (**H**) were supplemented with CuNPs (6.5 or 13 mg/kg from Cu-nanoparticles) together with cellulose (2%) and psyllium (6%) as a source of dietary fiber. A comparison of dietary fiber in regard to a standard (6.5 mg/kg) (**I**); and enhanced (13 mg/kg) (**J**) copper dose from CuNPs. Values are means ± SEM, n = 10, ^#^ vs. inulin, *p* ≤ 0.05 (two-way ANOVA with Tukey’s multiple comparisons test). COX inhibition potentiated vasodilation for SN vs. JN, and modified vasodilation for both JNH and SNH vs. PNH compared to arteries not incubated with COX-inhibitors.

COX inhibition (pre-incubation of the aortic rings with COX-1 and COX-2 inhibitors, SC-560 and NS-398, respectively) did not modify the vasodilator response compared to the control conditions for all studied groups. However, a significant difference was observed between JN and SN, see Figure 7A–J.

**Figure 8:**
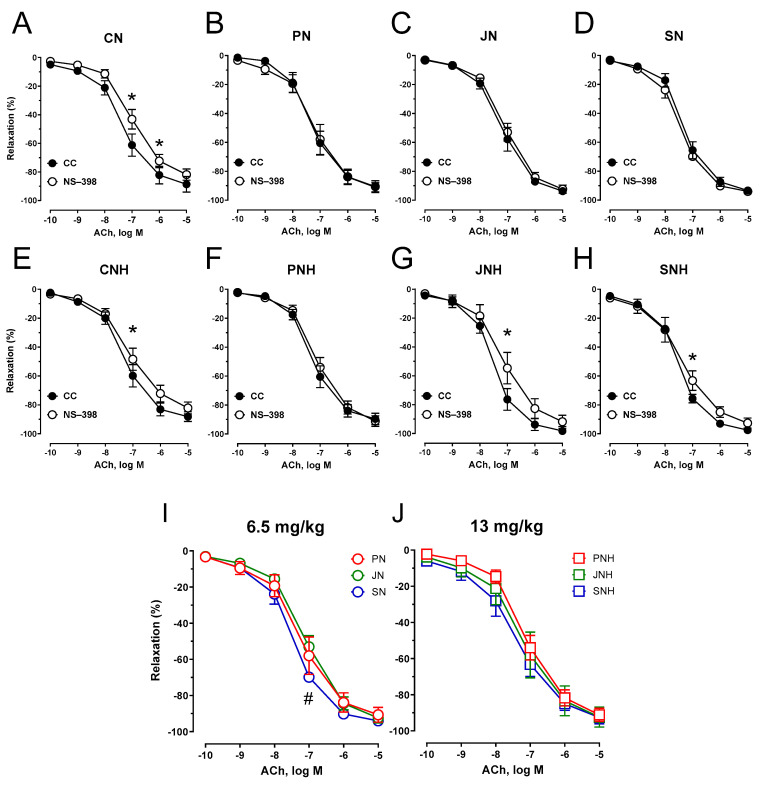
Effects of COX-2 inhibition on the cumulative concentration-response curves to acetylcholine in the isolated thoracic arteries from rats fed with experimental diets. Dietary treatments: rats from group CN (**A**) and CNH (**E**) were supplemented with CuNPs (6.5 or 13 mg/kg from Cu-nanoparticles) together with cellulose (8%) as a source of dietary fiber; group PN (**B**) and PNH (**F**) were supplemented with CuNPs (6.5 or 13 mg/kg from Cu-nanoparticles) together with cellulose (2%) and pectin (6%) as a source of dietary fiber; group JN (**C**) and JNH (**G**) were supplemented with CuNPs (6.5 or 13 mg/kg from Cu-nanoparticles) together with cellulose (2%) and inulin (6%) as a source of dietary fiber; group SN (**D**) and SNH (**H**) were supplemented with CuNPs (6.5 or 13 mg/kg from Cu-nanoparticles) together with cellulose (2%) and psyllium (6%) as a source of dietary fiber. A comparison of dietary fiber in regard to a standard (6.5 mg/kg) (**I**); and enhanced (13 mg/kg) (**J**) copper dose from CuNPs. Values are means ± SEM, n = 10, * vs. control conditions, ^#^ vs. inulin, *p* ≤ 0.05 (two-way ANOVA with Tukey’s multiple comparisons test). MDA, malondialdehyde. COX-2 inhibition did not attenuate vasodilation for JNH and SNH under an enhanced CuNP dose compared to the control conditions, as it did for the other groups.

Pre-incubation of the aortic rings with the selective COX-2 inhibitor (NS-398) attenuated the vasodilator response to acetylcholine in arteries from pectin (PN), inulin (JN), and psyllium (SN) compared to cellulose (CN) under the standard dose of CuNPs, see Figure 8A–D. This attenuation of vasodilation was also significant for pectin (PNH) under an enhanced CuNP dose, see Figure 8E–H. Neither inulin (JNH) nor psyllium (SNH) modified that response. COX-2 inhibition potentiated vasodilation for psyllium (SN) vs. inulin (JN), and modified vasodilation for both inulin (JNH) and psyllium (SNH) vs. pectin (PNH), see Figure 8I–J. Moreover, pectin (PN), inulin (JN), psyllium (SN), and PNH decreased the vasodilator net effect of COX-2-derived prostanoids in acetylcholine-induced vasodilation, see Figure 8A–H.

#### 3.3.2. ELISA Studies

Neither COX-1, iCAM-1, HO-1, GAPDH, nor eNOS were modified by dietary fiber (data not presented).

**Figure 9:**
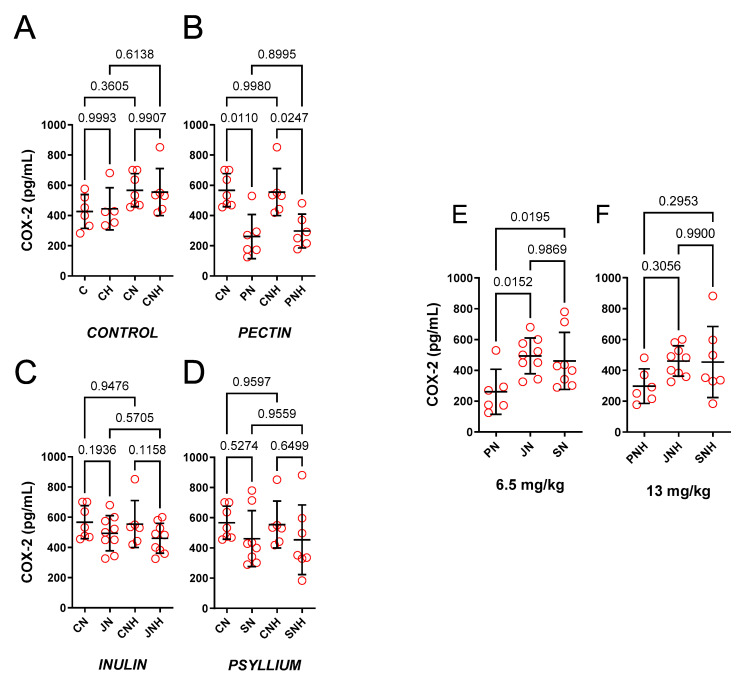
Cyclooxygenase-2 measured in the aortic rings of rats fed with experimental diets. Dietary treatments: rats from groups C and CH were fed a control diet with a standard and enhanced Cu content (6.5 or 13 mg/kg as CuCO_3_) together with cellulose (8%) as a source of dietary fiber (**A**); group CN and CNH were supplemented with CuNPs (6.5 or 13 mg/kg from Cu-nanoparticles) together with cellulose (8%) as a source of dietary fiber (**A**); group PN and PNH were supplemented with CuNPs (6.5 or 13 mg/kg from Cu-nanoparticles) together with cellulose (2%) and pectin (6%) as a source of dietary fiber (**B**); group JN and JNH were supplemented with CuNPs (6.5 or 13 mg/kg from Cu-nanoparticles) together with cellulose (2%) and inulin (6%) as a source of dietary fiber (**C**); group SN and SNH were supplemented with CuNPs (6.5 or 13 mg/kg from Cu-nanoparticles) together with cellulose (2%) and psyllium (6%) as a source of dietary fiber (**D**); a comparison of dietary fiber with a standard (6.5 mg/kg) Cu dose (**E**) and enhanced (13 mg/kg) Cu dose in the form of CuNPs (**F**). Under the standard dose of CuNPs, pectin (PN) decreased COX-2 compared to cellulose (CN), inulin (JN), and psyllium (SN). Under an enhanced dose of CuNPs, pectin (PNH) decreased COX-2 compared to cellulose (CN). Values are means ± SD, n = 10, *p* ≤ 0.05 (two-way ANOVA with Tukey’s multiple comparisons test). COX, cyclooxygenase.

The effect of a standard or an enhanced dose of Cu and nano-Cu (6.5 or 13 mg/kg) was insignificant, see Figure 9A.

*The effect of decreased cellulose content and high CuNP dose.*

PN (0.46-fold) and PNH (0.54-fold) decreased COX-2 compared to CN and CNH under standard and enhanced doses of CuNPs, see Figure 9B–D.

*The effect of PN* vs. *JN vs. SN and a high CuNP dose.*

PN decreased COX-2 compared to JN (0.53-fold) and SN (0.57-fold), see Figure 2E. Enhanced doses of CuNPs made this insignificant (0.65-fold and 0.66-fold, respectively), see Figure 9F.

## 4. Discussion

Ionic copper and zinc particles regulate cardiovascular system function [16]. We studied the effects of a standard (6.5 mg/kg diet) and a two-times higher dose of Cu in the form of nanoparticles (13 mg/kg of diet) with a combination of cellulose, pectin, inulin, and psyllium. These examinations were conducted with the implementation of functional studies on isolated rat hearts and thoracic arteries, as well as with the measurement of antioxidant status in hearts, aortic rings, and blood plasma. Male Wistar Han rats at 9 weeks of age were supplemented for another 6 weeks with 8% of added cellulose as a dietary fiber source (control), while the experimental fiber preparations (pectin—viscous effect, inulin—prebiotic effect, psyllium—bulk effect) were added at 6% in place of cellulose content. Dietary composition and treatments used in the experimental feeding period were previously thoroughly described by Cholewińska et al. [14].

### 4.1. High CuNP Effects

Oxidative stress induces functional changes in arteries [17,18,19]. Recent studies point out that metal nanoparticles in the diet influence oxidative stress, which modifies the vascular response. Chromium nanoparticles were recently studied in rats fed with a high-fat, low-fiber diet, and a negative impact on the antioxidant status and vasodilation of thoracic arteries was established [15]. Moreover, supplementation with CuNPs exacerbates the negative changes induced by hypertension in the vasculature, heart, liver, and intestines [14,20], and influences the oxidative stress markers, which modulate the vasodilation of thoracic arteries with nitric oxide and prostanoids involved in normotensive rats [21,22,23]. In addition, CuNPs also potentiate vascular contraction induced by prostaglandin F2-alpha. In the same study, the blood plasma Cu-to-Zn ratio was decreased due to a decreased Cu content [24]. Interestingly, the administration of fish oil elicited a favorable response in the vascular system of rats fed with an ionic Cu rather than CuNPs. As a possible mechanism, we pointed to increased SOD, an enzyme which catalyzes the dismutation of superoxide (O_2_^•−^), generating hydrogen peroxide (H_2_O_2_), decreased CAT (which converts harmful H_2_O_2_ into water), and decreased Cu concentration in the blood of rats supplemented with FO and CuNPs [25].

In the presented study, increasing the dose of CuNPs from 6.5 to 13 mg Cu/kg of diet had no effect on vascular relaxation nor the antioxidant status measured in hearts, aortic rings, and blood plasma. After pre-incubation of thoracic arteries with COX-2 inhibitor, this two-fold increase in the dose decreased the participation of vasodilator prostanoids in the acetylcholine-induced response, which points to a decreased production or sensitivity to COX-2-derived products. We have previously concluded that decreased participation of vasodilator prostanoids derived from COX-2, together with the increased participation of vasoconstrictor 20-hydroxyeicosatetraenoic acid (20-HETE) and the thromboxane receptors (TP), is the mechanism of a standard dose of CuNPs in Wistar rats [13].

### 4.2. The Effect of Decreased Cellulose Content and High CuNP Dose

#### 4.2.1. Sticky Pectin vs. Insoluble Cellulose

The most significant effect of viscous pectin was related to decreased CAT in the hearts of rats supplemented with CuNPs under both standard and enhanced doses. CAT is an important enzyme that takes part in the decomposition of H_2_O_2_ to water and oxygen, and it protects from oxidative damage induced by reactive oxygen species (ROS). H_2_O_2_ reacts with redox-active metals (e.g., iron) and generates the hydroxyl radical (^•^OH) through the Fenton/Haber-Weiss reaction. It must be said that CuNPs under the standard dose of Cu increased CAT, and that pectin decreased this effect. Although CAT was not modified by the enhanced CuNP dose, the pectin effect was also significant, which decreased CAT. In the vasculature, the implementation of pectin as a dietary fiber decreased the sensitivity/production of vasodilator prostanoids derived from COX-2, and this was visible under the standard and enhanced doses of Cu from CuNPs. This was confirmed by decreased COX-2 in the aortic rings of these rats. Cholewińska et al. previously observed that, among all the fiber preparations tested in this study, pectin, along with cellulose, inulin, and psyllium, has the greatest ability to inhibit pro-inflammatory processes, as pectin decreased caspase-3 in the wall of the small intestine [14].

#### 4.2.2. Prebiotic Inulin vs. Insoluble Cellulose

Under the standard dose of Cu from CuNPs, non-viscous inulin, with a significant prebiotic importance [14], increased the antioxidant capacity of lipid-soluble compounds (ACL) in the blood plasma, which corresponded with decreased oxidative stress reflected as decreased malondialdehyde (the frequently used oxidative stress biomarker) and CAT in the hearts and decreased participation of COX-2 derived vasodilator prostanoids in vascular relaxation. The vascular effect was similar to that described for pectin; however, the COX-2 amount was not modified as determined by ELISA assay. Under an enhanced dose of CuNPs, we observed increased vasodilation in response to acetylcholine due to COX-2 up-regulation (opposite to pectin, where COX-2 was down-regulated) and an increased effect of COX-1-derived prostanoids. Similar to the standard dose, CAT in the hearts was also decreased; however, the effect of malondialdehyde was insignificant. These changes might be explained by the viscous effect of pectin, which forms a sticky gel in contact with water, whereas non-viscous inulin is of significant prebiotic importance, which modulates the pH of the intestine and modifies the absorption processes mentioned in the introduction [14]. In the same study, the authors pointed to some beneficial effects of inulin on DNA repair mechanisms in the small intestine in contrast to either pectin or psyllium.

#### 4.2.3. Swelling Psyllium vs. Insoluble Cellulose

The effect of sticky, swelling psyllium with a bulk effect was similar to that of non-viscous prebiotic inulin under the standard dose of Cu from CuNPs. We observed an increase in the antioxidant capacity of lipid-soluble compounds in the blood plasma and decreased oxidative stress biomarkers (malondialdehyde and CAT) in the hearts, together with decreased sensitivity/participation of COX-2-derived vasodilator prostanoids in arterial relaxation. Under the enhanced dose of CuNPs, we observed an increased vasodilator response to acetylcholine (a similar effect to that induced by prebiotic inulin). However, the possible mechanism related to the observed changes in vasodilation was through the up-regulation of iNOS. Surprisingly, psyllium did not decrease CAT (measured in rats’ hearts), contrary to pectin or inulin. This may, at least in part, be explained by the fact that supplementation with either inulin or psyllium decreased daily dietary intake together with daily weight gain, which is not dependent on the dose of CuNPs used [14]. In the same study, the authors noticed a reduced dose of diamine oxidase (DAO), an indicator of intestinal integrity in the blood plasma of rats supplemented with inulin and psyllium, and a higher dose of zonula occludens-1 gene expression (a marker of intestinal inflammation and neoplastic lesions) for psyllium, exclusively [26,27]. Since DAO is an enzyme that catalyzes the decomposition of histamine, the above-mentioned results point to reduced inflammatory processes due to the addition of inulin and psyllium.

### 4.3. Pectin vs. Inulin vs. Psyllium

Under the standard dose of Cu from CuNPs, both inulin and psyllium were more beneficial compared to pectin, as these fiber products increased the antioxidant capacity of water- and lipid-soluble compounds in the blood plasma and decreased oxidative stress (decreased malondialdehyde) in the hearts of supplemented rats. Also, in our previous studies changes to ACW, ACL and malondialdehyde, by supplementation with various food products, were found to be beneficial to rats [15,28]. Surprisingly, this two-fold increase in the dose of CuNPs compared to dietary recommendations made this insignificant, which is hard to explain.

The most significant effect was related to CAT, and H_2_O_2_ decomposition into water and oxygen. We observed CAT being decreased for pectin compared to inulin and psyllium, and this was significant for both standard and enhanced doses of Cu from CuNPs. What’s more, the enhanced dose of CuNPs decreased CAT further for inulin compared to psyllium, which might, at least in part, explain the observed changes between these two groups of fiber preparations. Moreover, inulin and psyllium potentiated a vasodilator response of isolated aortic rings to acetylcholine compared to pectin, and this was only significant for the enhanced CuNP dose. When iNOS was blocked, it was psyllium which weakened vasodilation to acetylcholine compared to inulin under an enhanced CuNP dose. It must be mentioned that no significant change was observed due to iNOS inhibition for either pectin or inulin. Under the standard dose of Cu from CuNPs, we observed no difference in vasodilation between pectin and psyllium; however, inulin exhibits a tendency to decrease vasodilation of aortic rings preincubated with iNOS. Since up-regulation of iNOS contributes to the increased synthesis of COX-derived prostanoids, we examined the involvement of COX-1 and COX-2 pathways in acetylcholine-induced vasodilation. Under a standard dose of CuNPs, when both enzymes were blocked, a significant difference occurred between inulin and psyllium, which was not observed in aortic rings not incubated with COX-inhibitors (control conditions). This points towards the participation of COX-derived prostanoids in the vascular relaxation of rats fed with different types of dietary fiber. When the dose of CuNPs was enhanced, this became insignificant, which is a similar effect to the control conditions. However, COX inhibition made insignificant observed changes between pectin and the other two groups (inulin and psyllium) under the control conditions, pointing towards the significant effect of prostanoids in vascular relaxation on fiber-supplemented rats.

Our next step was to analyze which isoform was responsible for the described changes. Under the standard dose of Cu from CuNPs, COX-2 inhibition did not modify the observed changes described for COX inhibition (COX-1 plus COX-2), which suggests that pectin, inulin, and psyllium are responsible for the decreased participation of COX-2-derived prostanoids in vascular relaxation, and this was more pronounced for inulin. Under the enhanced dose of Cu from CuNPs, pectin decreased sensitivity to COX-2-derived prostanoids, contrary to inulin and psyllium, where it was potentiated. This points towards COX-2 up-regulation in vascular relaxation under the enhanced CuNP dose for inulin and psyllium and to the engagement of a vasoconstrictor compensatory mechanism(s) dependent on COX-1-derived prostanoids.

Surprisingly, in this study, heart SOD was neither modified by the CuNPs nor by the dietary fiber. SOD enzymes catalyze the dismutation of O_2_^•−^ generating H_2_O_2_. The reaction between O_2_^•−^ and nitric oxide (NO^•^) produces peroxynitrite (ONOO⁻), whose decomposition, in turn, gives rise to other highly oxidizing intermediates. Another mechanism of ROS formation involves O_2_^•−^ protonation, which gives rise to reactive hydroperoxyl radical (HO_2_^•^). Moreover, SOD is dependent on the level of Cu and Zn, so any fluctuations in the dietary concentration of these elements may change SOD activity. Since SOD was not modified, we concluded that the increased CuNP dose did not modify the equilibrium between O_2_^•−^ and H_2_O_2_, and that dietary fiber did not provide protection against ROS derived from O_2_^•−^. Also, ELISA assays performed for COX-1, HO-1, NOS3, GAPDH, and ICAM-1 detection, which are the markers of an inflammatory state, did not provide significant information about these processes in the body opposite to the COX-2 assay. As the systolic, diastolic, mean arterial pressure, and heart rate in the Langendorff heart were not modified, we found it rather beneficial, since dietary products, under typical daily doses, should not modify the heart’s functioning in contrast to cardiac medications, which are prescribed for selective heart disorders and are not free of side effects.

## 5. Conclusions

Dietary implementation of CuNPs, in place of ionic Cu, reduced the antioxidant capacity of lipid-soluble compounds of blood plasma and increased H_2_O_2_ decomposition in hearts, while having no effect on either the vasodilation of isolated aortic rings or heart O_2_^•−^ dismutation (SOD). When the dose of CuNPs was increased from 6.5 to 13 mg Cu/kg of diet, it had no negative effect on the antioxidant status or the vascular response. The most visible effect on antioxidant mechanisms and vasodilation are related to inulin and psyllium. However, pectin had the greatest ability to inhibit the production of prostanoids derived from COX-2 in aortic rings, which highly modified the H_2_O_2_ decomposition in hearts. This beneficial effect of dietary fiber modulates the antioxidant mechanisms, which protect the vasculature and the heart against potentially harmful CuNPs.

## Data Availability

Data are available on request.

## Supplementary Materials

The following supporting information can be downloaded at: https://www.mdpi.com/article/10.3390/nu15163557/s1, Table S1: The ELISA kits used for the determination of antioxidant status in the presented study.
