# Supplementary material for: The Interaction of Dietary Pectin, Inulin, and Psyllium with Copper Nanoparticle Induced Changes to the Cardiovascular System
Source: Nutrients. 2023 Aug 11;15(16):3557. doi: 10.3390/nu15163557 (PMC10457830; doi:10.3390/nu15163557)
Supplement: Supplementary file 1 [file nutrients-15-03557-s001.zip › nutrients-2528889-supplementary.pdf]

Table S1. The ELISA kits used for the determination of antioxidant status in the presented study

	Antigen	ELISA kit catalogue number	Manufacturer, country	Assay range (pg/ml)
1.	Rat PTGS1 ELISA Kit	orb566784	Biorbyt, UK	0.156–10 ng/mL<br>Intra-Assay: CV<8%<br>Inter-Assay: CV<10%
2.	Rat PTGS2 ELISA Kit	orb1199748	Biorbyt, UK	78.1–5000 pg/mL<br>Intra-Assay: CV<10%<br>Inter-Assay: CV<12%
3.	Rat HO-1 ELISA Kit	orb567452	Biorbyt, UK	0.156–10 ng/mL<br>Intra-Assay: CV<8%<br>Inter-Assay: CV<10%
4.	Rat NOS3 ELISA Kit	orb566738	Biorbyt, UK	15.625–1000 pg/mL<br>Intra-Assay: CV<8%<br>Inter-Assay: CV<10%
5.	Rat GAPDH ELISA Kit	orb567887	Biorbyt, UK	0.156–11 ng/mL<br>Intra-Assay: CV<8%<br>Inter-Assay: CV<10%
6.	Rat ICAM-1 ELISA Kit	RAB0221-1KT	Sigma-Aldrich, Germany	28.81–7000 pg/mL<br>Intra-assay cv: <10%<br>Inter-assay cv: <12%
